# Some Suggestions Regarding the Management of Hysterical Disorders

**Published:** 1887-08

**Authors:** Edward B. Angell

**Affiliations:** Rochester, N. Y.


THE BUFFALO Medical AND Surgical Journal

VOL. XXVIII. AUGUST, 1887. NO. 1.

### Original Communications.

### *SOME SUGGESTIONS REGARDING THE MANAGEMENT OF HYSTERICAL DISORDERS.*

By EDWARD B. ANGELL, M. D., of Rochester, N. Y.

(Read before the Central New York Medical Association.)

The growing complexity of our social life, with its struggles for pre-eminence, its unceasing demands and constant tension, no doubt has had much to do with the rapid increase of that large class of nervous disorders, of which hysteria forms a predominant feature.

The common hysterical fit is, perhaps, its simplest and most fleeting expression, but it is rather the obscurer and more prolonged manifestations of the hysterical temperament with which this paper deals.

Whatever may prove to be the organic basis of this functional disorder, one peculiar psychical condition is prominent in nearly every case, a curious inco-ordination of the mental faculties. While the will is not lost, its action is materially affected. As Sir James Paget admirably remarks: "They say 'I cannot;' it looks like 'I will not;' but it is 'I cannot will.'" This ataxy of the will may result from several distinct causes, among which, as more important, may be suggested exaltation of the sensory tract, developing exaggerated ideas of peripheral irritation. In this way pain, which ordinarily would be of little moment, becomes unbearable; the process of digestion, usually unnoticed, attracts attention and gives rise to uneasy or distressing gastric sensations, often confounded, no doubt, with real dyspeptic symptoms. The abdominal pulsations, the throbbings and beatings complained of elsewhere are, doubtless, due to this extreme sensitiveness to normal physical conditions, of which, ordinarily, we are wholly unconscious. Again, the consciousness, itself abnormally keen, may exaggerate and misconstrue sensations which are, themselves, perfectly natural. We all know the power of expectant attention, and the heightened sensitiveness it develops. Or the emotional nature has been allowed to run riot till it overtops the judgment, and all ideas are subject to its wayward caprice. With the breaking down of the controlling influence of the will, the whole being responds more immediately to the impulses sent upward from the lower nervous centers, and ideas, emotions, thought, even, and their active expression, approximate more nearly the reflex process, the patient becoming a pitiable automaton.

The psychological aspect of hysteria has been thus made prominent, since therein, in great measure, must we find explanation of the general futility of drugs in its treatment. Says Dr. Weir Mitchell, in his very suggestive work on "Nervous Diseases": "Nothing, I think, can be more melancholy than an honest survey of the amount of good done in hysteria by the host of drugs which go to form the so-called therapeutics of this disease." The varying expressions of the malady, simulating, as it does, nearly every organic affection of the nervous system, are but symptoms, and failure to cure often comes through failure to treat the real malady.

Grasset, in a recent number of *Brain*, speaking of the treatment of this disease, says: "Means of treating the paroxysm, of removing anæsthesia, of combatting single symptoms, are, perhaps, to be found in abundance, but the groundwork of the disease, the neurosis or morbid state, is not attacked."

It is rather with reference to this point that this paper was written, while but slight allusion will be made to the therapeutical routine ordinarily employed in combatting the disease.

Toward the attainment of success in treatment, nothing is more urgent than a careful attention to the diagnosis. Trite as this observation must seem, it is of all importance in the management of the case. A most careful study of the symptoms, a searching investigation for undeniable evidences of organic mischief must be made; for when once the conclusion has been reached that the malady is purely hysterical, no assertion of new and strange symptoms must be allowed to shake the judgment or affect the purpose. Just here, homeopathy, with its certain remedy for every complaint, fails utterly, its therapeutics following ever a fleeting phantom. With the diagnosis, then, well established, determine upon the course of treatment to be pursued, and carry it out steadily and persistently, confident of ultimate success, undeterred by the intervention of fortuitous symptoms. To succeed in this, the gaining of your patient's confidence is of greatest moment. Study her character, her surroundings, her likes and dislikes, her social relations, for they all have much to do with establishing the hysterical temperament.

For this end nothing avails more than a careful, considerate estimation of her troubles. Show her you recognize the reality of her suffering, the gravity of her condition. Do not commit the error of making light of her fears and fancies as whims of the imagination; for however unreal they are from an objective standpoint, to her they have intense reality, and are undeniably significant. It matters little whether abnormal sensations arise from evident pathological conditions or from perturbed nervous action, the result, so far as the patient's consciousness is concerned, must be the same, with this exception: pain and distress from organic disease is far more wearing than the severest suffering of functional origin. Usually the hysterical subject experiences far less wear and tear than do the sympathizing friends about her. I have seen a case of hystero-epilepsy endure for hours severe muscular contortions with little subsequent fatigue, while her attendants would be utterly worn out. Indeed, this lack of physical friction constitutes one of the marked features of this curious disease. But while considerate in all your relations, firmness in dealing with the crotchets and fancies is yet more essential. Remember, she will watch you narrowly to discover if they cause you anxiety, for hysteria has a morbid craving to perplex—instability is its nature, and it likes company. But when you do gain the mastery, when by thorough appreciation of the suffering you secure confidence, and by unruffled purpose you inspire belief, through this very lack of self-stability will come rest, re-assurance and hope—three very potent agents for good.

One of the most serious obstacles the physician has to deal with is the mistaken sympathy and over-anxiety of friends. In nervous affections of this type, often a patient's friends are her worst enemies, for their very anxiety and unceasing attention do much to feed and stimulate the emotional sensibilities you would seek to curb. All-important, then, particularly in the worst cases, is isolation. Its necessity in these cases cannot be too strongly emphasized. You break up thereby at once a whole train of evils, and bring a new set of influences to bear with powerful effect upon habits which, amidst familiar surroundings, had become well-nigh confirmed. New scenes, strange faces, rigid adherence to routine, are not congenial, and usually the first week of isolation, with a stranger for a nurse, shows a definite gain in self-control that comes through a feeling of restraint. Rarely can this advantage be secured in the patient's home. It is best obtained by removal to the seclusion afforded by a well-regulated hospital. The absence of sympathetic friends, the lack of hurtful excitement, the steady daily routine, go far toward discouraging attention to the physical vagaries which had been so exacting of attendant and patient alike. Under the influence of novel surroundings, the dormant will is aroused and opportunity secured to give wholesome suggestion and advice, if your patient chance to be intelligent and apprehensive.

Hysteria thrives best on a poor soil. In fact, we meet with very few cases that do not bear plain evidence of mal-nutrition.

The need of tonics, of fresh air, of good feeding, is well enough recognized. Its importance cannot be too thoroughly dwelt upon. But the place of nutrition is so well established, I need only refer to it here. To secure that, there is no better means than the measures first brought to notice by Dr. Mitchell and used by him with such success. The combination of rest with high feeding, massage and electricity, will make flesh and blood when all other means fail; and with improving color and weight there will come a corresponding elevation of the moral tone. I shall say little of the possible reflex causes of hysteria.

They must be sought for and removed, if possible. Yet there is need of caution in one direction particularly. Pelvic irritation may be due to over-sensitive nerve centers quite as well as to some uterine irregularity.

I do not find it serviceable to make light of the symptoms or call them imaginary ills. That would bring distrust and antagonism. Rather explain how readily the consciousness misinterprets the sensations conveyed to it by an over-sensitive nervous system, thereby exaggerating their nature and significance. Or again, emphasize the strong influence self-attention has had in accenting slight disturbances, and show how the unrestricted play of the emotional nature has intensified the susceptibilities till the judgment is warped and unable to form just conclusions regarding subjective sensations. In some such way give your patient a clear insight into the real nature of her despotic malady, the need of disregarding its manifold manifestations, and, above all, the great advantage of steadying purpose. Toward gaining this the patient must avoid, as far as possible, the evil habit of introspection, of analyzing symptoms and finding causes. She must be kept pretty constantly busy, with little opportunity for morbid self-study. The duties of the day must be such as to occupy the mind, but not weary it. Trifles, which require the exercise of a slight amount of will power, to be increased as capacity for definite application is gained, will be of service with the improving strength. But care must be exercised lest the moral and physical strength be not over-tasked. Nothing will discourage more than exhaustion, and all required effort must stop short of that. Many times you will meet with obstinacy, or, worse, morbid perversity. You may be made the target for the venting of personal malice and spite, and rarely will the physician experience a more trying test of self-control than such an encounter affords. Yet at no time is the opportunity for good greater, since an unruffled demeanor will have marked influence in cutting short such emotional paroxysms. I will admit that, under certain provocation, an assumption of high-minded indignation may be called for, but it is a weapon requiring utmost care in its use. An emotional nature is very strong for a brief period, but it is quickly exhausted and cannot withstand the power of steady, undisturbed purpose. Direct command has usually little weight. Rather win your battles by tact and skill. Encourage effort, praise success, be patient with failure.

Suggestion is a power if coupled with approval over honest effort, however successful. It should find expression not alone by word but by conduct as well. The hysterical mind acutely conscious of her own sensations is very watchful of expression and action on the part of those about her. Be wary of showing surprise or anxiety over new symptoms. Assiduous attention to her varying tale of ailments but feeds an exacting appetite. We all know how dearly many women love to dwell upon and discuss their aches and ills with each other, and with the hvsterical this morbid craving for personal sympathy becomes a passion. So far as possible, this same disregard of fugitive symptoms must enter into the treatment. Here judicious neglect will often cure, where direct attention might but add importance and establish persistence. This need of consistent effort in one direction, looking toward the cure of the essential malady rather than the relief of its symptoms, cannot be too strongly commended, for the sake of the suggestive influence such a course has upon the patient herself. If, on the contrary, you let her find she can sway your judgment at will by constant and varied complaints, your influence is gone, for no sooner will one symptom be conquered ere a new expression will demand attention, and your list of antispasmodics, analgesics and alteratives, extensive as it is, will speedily be exhausted. Indeed, it is far safer to disregard entirely all subjective symptoms and be guided only by objective signs, the result of careful investigation from time to time. She complains of terrible distress in her stomach, possibly regurgitates her food, but if the stools are fairly regular and indicative of good digestive action, while her body weight only alters for the better, be not deceived by her complaints, for in due time they will be forgotten, killed by intentional neglect.

A few words need be said about the nurse, for she plays a most important part. She requires tact, discretion, firmness, and the rare ability to execute orders with little attendant fuss. She should be attractive both in appearance and bearing, and should command the respect as well as win the good will of her patient. If officious or too familiar, if impatient or too slow, she will usually prove unsatisfactory, and a change had best be made; for a poor nurse is as mischievous as a good one is helpful. It is manifestly wiser, in general, to discuss progress and give orders aside, as the patient is keenly sensitive to derogatory remarks or partially-understood directions; while few nurses would jeopardize their comfort by giving unvarnished reports in her presence.

Little need be said regarding the use of drugs, nor can much be asserted of their value, except as aids to the general nutrition or makeshifts to cover too clamorous symptoms. Their sphere is largely secondary, and choice may be made according to the particular need. He who relies upon their efficacy and exhausts his mathematical skill in arranging their permutations and combinations will usually fail, at least, of that larger success the true physician works for—the ultimate cure of the morbid nature. But with time, that very important factor in the treatment of all nervous ailments, with improved nutrition from careful but generous diet and efficient though not fatiguing exercise, with the aid of a well-trained nurse, and, above all, with a skillful application of moral force, will you not only emancipate your patient, but you will teach her to know herself and her disease, how to care for the one and avoid the other.

